# Obtaining Eggs from Xenopus laevis Females

**DOI:** 10.3791/890

**Published:** 2008-08-20

**Authors:** Marie K. Cross, Maureen Powers

**Affiliations:** Department of Cell Biology, Emory University

## Abstract

The eggs of Xenopus laevis intact, lysed, and/or fractionated are useful for a wide variety of experiments. This protocol shows how to induce egg laying, collect and dejelly the eggs, and sort the eggs to remove any damaged eggs.

**Figure:**
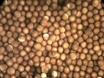


## Protocol

The complete text protocol for this experimental approach is available in Current Protocols in Cell Biology.

